# Assessment of tenecteplase target-associated pathogenic mechanisms underlying depression in acute ischemic stroke patients: insights from artificial intelligence-driven multi-omics analysis and *in vitro* validation

**DOI:** 10.3389/fnins.2026.1848128

**Published:** 2026-06-16

**Authors:** Qi Wang, Xihua Sun, Qiang Fu, Jiaqi Lv, Li Dong, Qi Yan

**Affiliations:** Neurology Department, FAW General Hospital of Jilin Province, Changchun, Jilin, China

**Keywords:** acute ischemic stroke, artificial intelligence, depression, multi-omics, tenecteplase

## Abstract

**Background:**

As a first-line treatment for acute ischemic stroke (AIS), tenecteplase (TNK) can cause adverse effects, such as depression, in AIS patients.

**Objective:**

This study aims to elucidate the TNK target-related pathogenic mechanisms underlying major depressive disorder (MDD) in AIS patients.

**Methods:**

By analyzing six public peripheral blood bulk datasets from AIS and MDD patients using integrative bioinformatics methods (limma, non-negative matrix factorization (NMF), and machine learning), we identified TNK target-associated molecular subgroups and diagnostic models for MDD and AIS patients, respectively. Next, a hub gene involved in the pathogenesis of both MDD and AIS was identified, and its corresponding molecular characteristics were analyzed in the peripheral blood bulk profiles of MDD and AIS patients. In addition, to gain a deeper understanding of the molecular implications of the hub gene involved in the pathogenesis of MDD in AIS, we performed disease ontology (DO) analysis and virtual cell knockout (KO) of the hub gene using public AIS mouse brain single-cell datasets. Furthermore, a deep learning pipeline (DrugReflector) model and molecular docking were used to identify MDD-preventive therapeutic agents for AIS patients based on MDD and AIS public blood bulk data. Finally, the expression pattern of the hub gene was also evaluated in MDD and AIS cell models.

**Results:**

Myeloperoxidase (MPO) can be considered an upregulated TNK target-associated gene involved in the pathogenesis of MDD in AIS patients, and BRD-K11973162 can be considered an MDD-preventive therapeutic candidate for AIS patients after TNK treatment.

**Conclusion:**

Our study is the first to identify MDD-associated diagnostic and therapeutic candidates for AIS patients after TNK treatment, providing a novel strategy for their clinical management.

## 1 Introduction

Acute ischemic stroke (AIS), resulting from the occlusion of cerebral blood vessels, is a leading cause of morbidity and mortality worldwide (Ho and Powers, 2025). Its pathophysiology involves a rapid sequence of events, including energy failure, excitotoxicity, oxidative stress, and intense neuroinflammation, culminating in irreversible neuronal damage (Walter, 2022). Intravenous thrombolysis with tenecteplase (TNK), a genetically engineered variant of tissue plasminogen activator, represents a first-line treatment for restoring cerebral blood flow (Warach et al., 2020). While effective in reducing infarct size, TNK therapy is not without risks (Alamowitch et al., 2023). Complications can include symptomatic intracranial hemorrhage, angioedema, and systemic bleeding events, which are well monitored (Ucar, 2019). In addition to these acute complications, a growing body of evidence points to long-term neuropsychiatric consequences that are less well understood.

Post-stroke depression (PSD) is a prevalent and debilitating complication that affects approximately one-third of AIS survivors, severely hindering cognitive and functional recovery (Lenzi et al., 2008). Intriguingly, clinical observations suggest that systemic inflammatory and reperfusion injury processes exacerbated by thrombolytic agents such as TNK may inadvertently elevate the risk of developing major depressive disorder (MDD) (Robinson and Jorge, 2016; Gupta et al., 2002). The abrupt reperfusion can amplify oxidative stress and trigger a robust inflammatory response, creating a milieu that may disrupt neurotrophic support and monoamine neurotransmission—key systems implicated in depression (Zhang and Liao, 2020). However, a precise molecular link between TNK pharmacodynamics and the pathogenesis of depression in AIS patients has not been established.

To address this critical gap, we conducted a comprehensive artificial intelligence (AI)-driven multi-omics investigation to elucidate PSD-related pathogenic patterns in AIS patients after TNK treatment. This study systematically identifies a key hub gene, validates its diagnostic utility, elucidates its cell type-specific role in the brain using single-cell genomics, and employs deep learning to discover a potential therapeutic agent, thereby providing a translational roadmap for mitigating depression in TNK-treated AIS patients.

## 2 Materials and methods

### 2.1 Source of datasets

Publicly available human peripheral blood transcriptomic datasets from AIS and major depressive disorder (MDD) patients were retrieved from the Gene Expression Omnibus (GEO) database using the GEOquery package in R, and they were normalized using the limma package in R (Davis and Meltzer, 2007; Ritchie et al., 2015). The TNK drug target gene set was curated from the Comparative Toxicogenomics Database (CTD) and is presented in Supplementary Table S1 (Davis et al., 2023). The datasets used in this study are listed in Table 1.

**Table 1:** Datasets used in this study.

ID	Platform	Purpose	Samples	Ref.
GSE58294	GPL570	Internal set 1	23 controls:69 AIS	Liu et al., (2022)
GSE32280	GPL570	Internal set 2	7 controls:8 MDD	Wu et al. (2024)
GSE16561	GPL6883	Training set 1	20 controls: 39 AIS	Chen et al. (2023)
GSE19738	GPL6848	Training set 2	34 controls: 33 MDD	Zou et al. (2023)
GSE22255	GPL570	Validation set 1	20 controls: 20 AIS	Zhang et al. (2024)
GSE23848	GPL6106	Validation set 2	15 controls: 20 MDD	Gu et al. (2025)

### 2.2 Identification of DEGs

Differential expression analysis between disease and control samples was performed separately for GSE58294 and GSE32280 using the limma package in R (Ritchie et al., 2015). Genes with a |log2FC| > 0.5 and a padj < 0.05 were considered significant differentially expressed genes (DEGs) (Zhou et al., 2026). The overlap between the AIS-DEGs and MDD-DEGs was identified, and the common DEGs were then overlapped with the TNK target gene list to obtain the final TNK-AIS-MDD (TAM)-associated DEGs. Their expression patterns in GSE58294 and GSE32280 were subsequently visualized using the ggplot2 package in R (Zhou et al., 2026). Functional enrichment analyses (KEGG and GO) were performed using clusterProfiler in R, based on KEGG and GO gene sets downloaded from the MSigDB database (Yu et al., 2012). A protein–protein interaction (PPI) network was constructed using the STRING database (confidence score > 1) and visualized using Cytoscape software (Szklarczyk et al., 2023).

### 2.3 NMF analysis

Non-negative matrix factorization (NMF) was applied separately to the expression matrices of TAM-associated DEGs in AIS samples from GSE16561 and MDD samples from GSE19738 using the NMF package in R (Sharma et al., 2020). The optimal number of clusters (k = 2) was determined by maximizing the cophenetic correlation coefficient (Sharma et al., 2020). GSEA was performed using the clusterProfiler package in R with KEGG and GO gene sets downloaded from MSigDB to compare pathway activities between the derived subgroups within each disease cohort. Immune cell deconvolution was performed using the CIBERSORT package in R to compare immune infiltration between the same subgroups (Yu et al., 2012; Chen et al., 2018).

### 2.4 Machine learning for diagnostic model construction

In each training set (GSE16561 for AIS and GSE19738 for MDD), a two-step feature selection strategy was applied to the TAM-associated DEGs. First, a random forest (RF) model using the randomForest package in R was employed to rank gene importance (Mean Decrease Gini) (Xu et al., 2023). The top-ranked genes were then subjected to Least Absolute Shrinkage and Selection Operator (LASSO) regression using the glmnet package (with 10-fold cross-validation) to identify the most robust, non-redundant hub gene (Zheng et al., 2023). Single-gene GSEA was performed to infer the biological functions of the hub gene in each context using clusterProfiler in R, based on KEGG and GO gene sets downloaded from the MSigDB (Yu et al., 2012). Diagnostic performance was evaluated using ROC and PR curves via the pROC package in R, and a nomogram with calibration curves was constructed using the rms package in R for the two training sets (Robin et al., 2011; Lin et al., 2024). Validation was performed on independent AIS (GSE22255) and MDD (GSE23848) datasets using ROC and PR curve analyses with the pROC package in R (Robin et al., 2011).

### 2.5 Single-cell analysis

The mouse brain scRNA-seq data (GSE174574, including three AIS samples) were processed using the Seurat package in R (Yu et al., 2026; Butler et al., 2018). Briefly, cells with unique feature counts <200 or >6,000 or mitochondrial gene content >20% were filtered (Butler et al., 2018). The data were then normalized, scaled, and clustered (Butler et al., 2018). Cell types were annotated using SingleR in R with a mouse brain reference (Aran et al., 2019). Intercellular communication was analyzed using CellChat in R (Jin et al., 2021). Metabolic heterogeneity was assessed using the SCENITH package in R (Argüello et al., 2020). Virtual knockout (KO) of the hub gene specifically within the astrocyte cluster was performed using the scTenifoldKnk package in R (FDR < 0.05) (Osorio et al., 2022). The top 10 perturbed genes associated disease phenotypes were analyzed via disease ontology (DO) enrichment using the DOSE package in R, and KEGG and GO pathway analyses were conducted using clusterProfiler in R based on KEGG and GO gene sets downloaded from the MSigDB (Yu et al., 2012; Schriml et al., 2022). Monocle 2 analysis in R was used for the investigation of targeted cell pseudotime trajectory patterns at the single-cell level (Huang et al., 2024).

### 2.6 Deep learning drug screening and molecular docking

The DrugReflector deep learning framework was applied independently to the gene expression profiles of GSE16561 (AIS) and GSE19738 (MDD) to screen the Connectivity Map (CMAP) database for compounds whose signatures could reverse the disease state (Ma, 2026). The intersection of the top candidates from both screens was then identified. Molecular docking between the final candidate compound (Pubchem CID: 6323266) and the protein structure of the hub gene (myeloperoxidase (MPO), PDB ID: 6BMT) was performed to evaluate binding affinity (kcal/mol). Briefly, molecular docking analysis was performed using AutoDock Vina (version 4.2.6) (Wang et al., 2022). The software was employed to predict potential binding pockets on the protein surface and to carry out flexible molecular docking, calculating docking scores and binding affinities (Vina score, in kcal/mol) for each pocket (Wang et al., 2022). The results were then ranked based on binding energy, and the pocket with the lowest binding energy was selected for further analysis (Wang et al., 2022). The binding modes and hydrogen-bond interactions were visualized in three dimensions using PyMOL (Wang et al., 2022). These visualizations clearly display key interactions, such as hydrogen bonds, hydrophobic contacts, and *π*–π interactions (Wang et al., 2022).

### 2.7 Cell lines, culture conditions, and qRT-PCR

The human SVGP12 cell line (ID: IM-H489) was purchased from Immunocell (China) and cultured in high-glucose Dulbecco’s Modified Eagle Medium (DMEM) (Gibco, United States) supplemented with 10% FBS (Gibco, United States) and 1% penicillin–streptomycin (Gibco, United States). To mimic AIS conditions, the cells were subjected to oxygen–glucose deprivation/reperfusion (OGD/R) for 4 h, followed by reperfusion. To model MDD-related stress, the cells were treated with 200 μM corticosterone for 24 h. Total RNA was extracted using TRIzol (Takara, China). The expression of MPO mRNA was quantified by qRT-PCR using SYBR Green (Vazyme, China) and normalized to GAPDH. The qRT-PCR primers (5′-3′) used in this study are listed below:

MPO:

F: GATCTGAGCTTCAACCCCCTG.

R: TCCACAGCCACCAGATTCTC.

GAPDH:

F: CCTTCATTGACCTCAACTACATGGT.

R: TCATTGTCATACCAGGAAATGAGCT.

### 2.8 Statistical analysis

For the bioinformatics analysis, an FDR of <0.05 or a two-sided *p*-value of <0.05 was considered statistically significant. For *in vitro* data, the results are presented as mean ± SD from at least three experiments. Comparisons were performed using an unpaired *t*-test. A *p*-value of <0.05 was considered statistically significant. Analyses were conducted using R (v4.2.2) or GraphPad Prism (version 9.0).

## 3 Results

### 3.1 Identification of TNK target-associated DEGs in AIS and MDD patients

**Figure 1:**
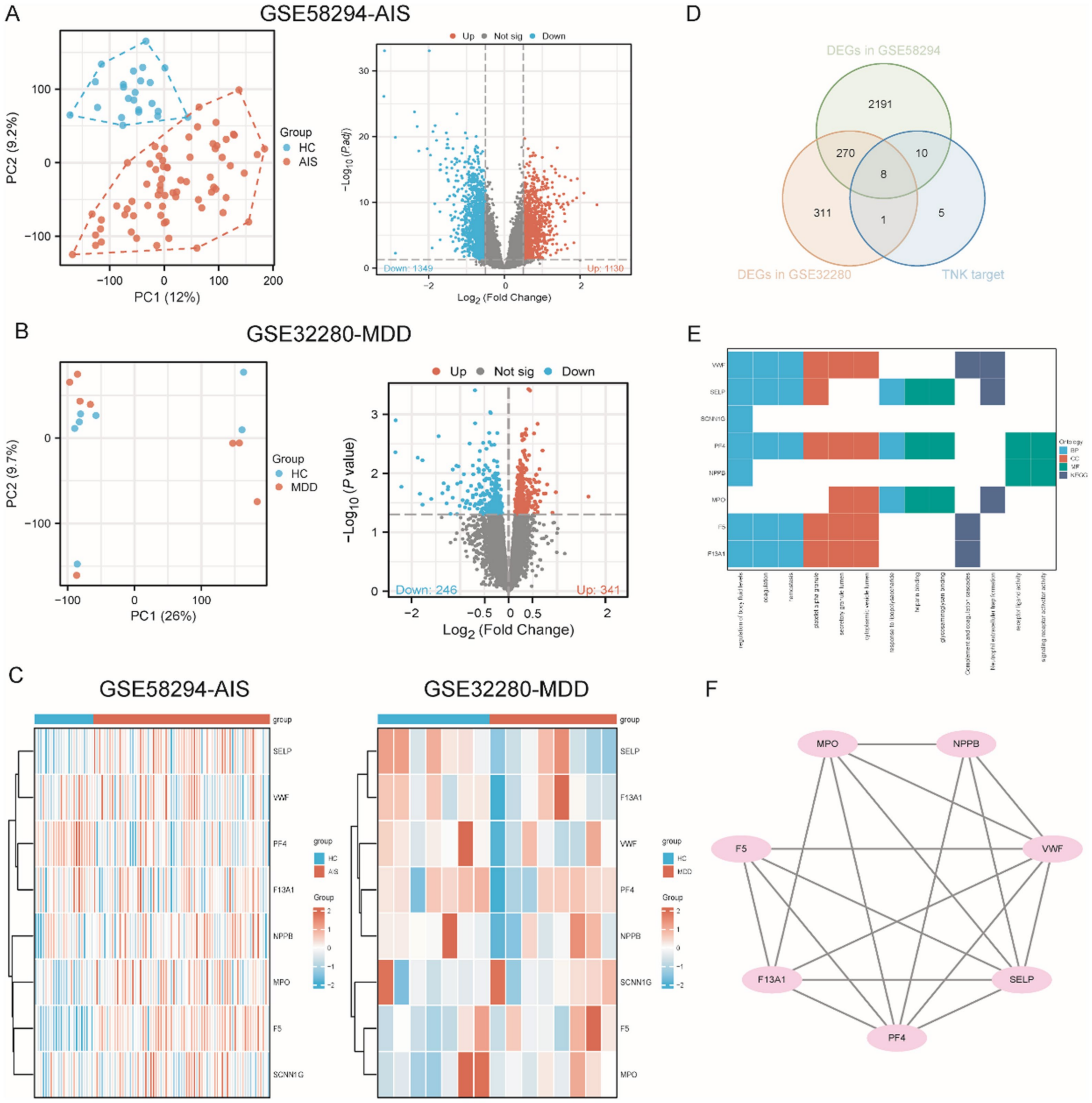
Identification and characterization of TAM-associated DEGs. **(A)** Identification of DEGs in GSE58294. **(B)** Identification of DEGs in GSE32280. **(C)** Heatmaps showing the expression patterns of the eight DEGs in GSE58294 and GSE32280. **(D)** Venn diagram showing the intersection of TAM-associated DEGs. **(E)** Bubble plot of the top enriched GO and KEGG terms for the eight TAM-associated DEGs. **(F)** PPI network of the eight TAM-associated DEGs constructed from the STRING database.

Differential expression analysis of the internal datasets identified significant DEGs in both the AIS (GSE58294) and MDD (GSE32280) cohorts (Figures 1A,B). The intersection of these DEGs with the TNK target list yielded eight TAM-associated DEGs (Figure 1D). KEGG and GO analyses revealed their involvement in functions related to neutrophil extracellular trap formation and reactive oxygen species metabolic processes (Figure 1E). Expression heatmaps confirmed the consistent dysregulation of these genes in both diseases (Figure 1C). The PPI network analysis demonstrated strong interactions among the eight genes (Figure 1F).

### 3.2 Identification of TNK target-associated molecular subgroups in AIS and MDD patients

**Figure 2:**
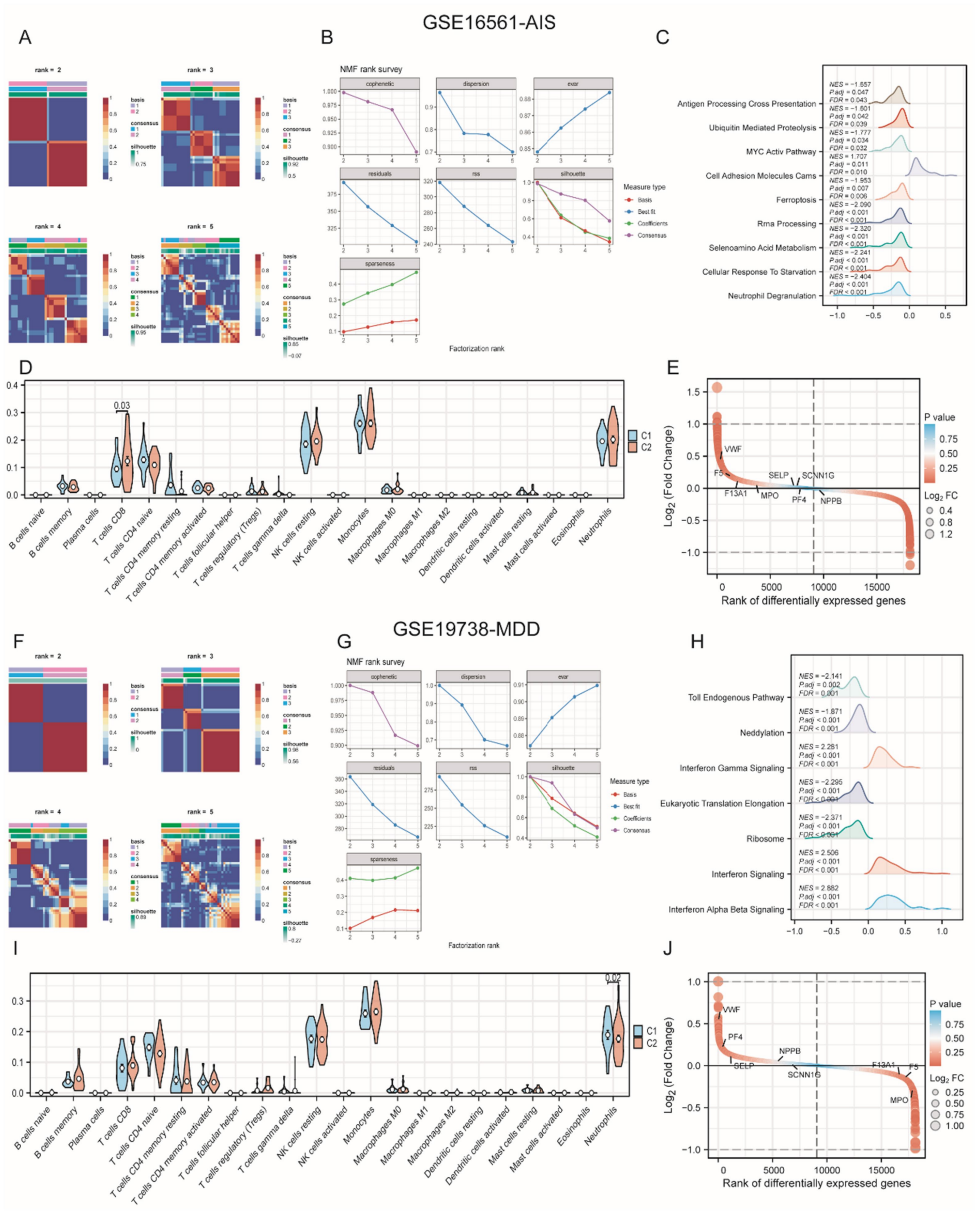
Molecular and immune heterogeneity of TNK-target-associated subgroups. **(A,B,F,G)** Consensus matrix heatmaps for NMF clustering (*k* = 2) in GSE16561 (AIS) and GSE19738 (MDD). **(C,H)** GSEA enrichment plots for GSE16561 (AIS) and GSE19738 (MDD). **(E,J)** Heatmaps comparing the average expression level of the eight TAM-associated DEGs between the C1 and C2 subgroups in both cohorts. **(D,I)** Box plots showing differential infiltration of selected immune cell types between the subgroups.

NMF consensus clustering based on the eight TAM-associated DEGs robustly stratified patients in both the AIS (GSE16561) and MDD (GSE19738) training sets into two subgroups (C1 and C2) (Figures 2A,B,F,G). In both diseases, the C2 subgroup exhibited significantly higher expression of all eight TAM-associated DEGs (Figures 2E,J). The GSEA results indicated that the C2 subgroup in both AIS and MDD datasets was enriched in inflammatory pathways (Figures 2C,H). Additionally, the CIBERSORT analysis revealed that the C2 subgroup had a distinct immune landscape, characterized by increased neutrophil and CD8 T-cell infiltration in MDD and AIS, respectively (Figures 2D,I).

### 3.3 Identification of the TNK target-associated hub gene in AIS and MDD patients

**Figure 3:**
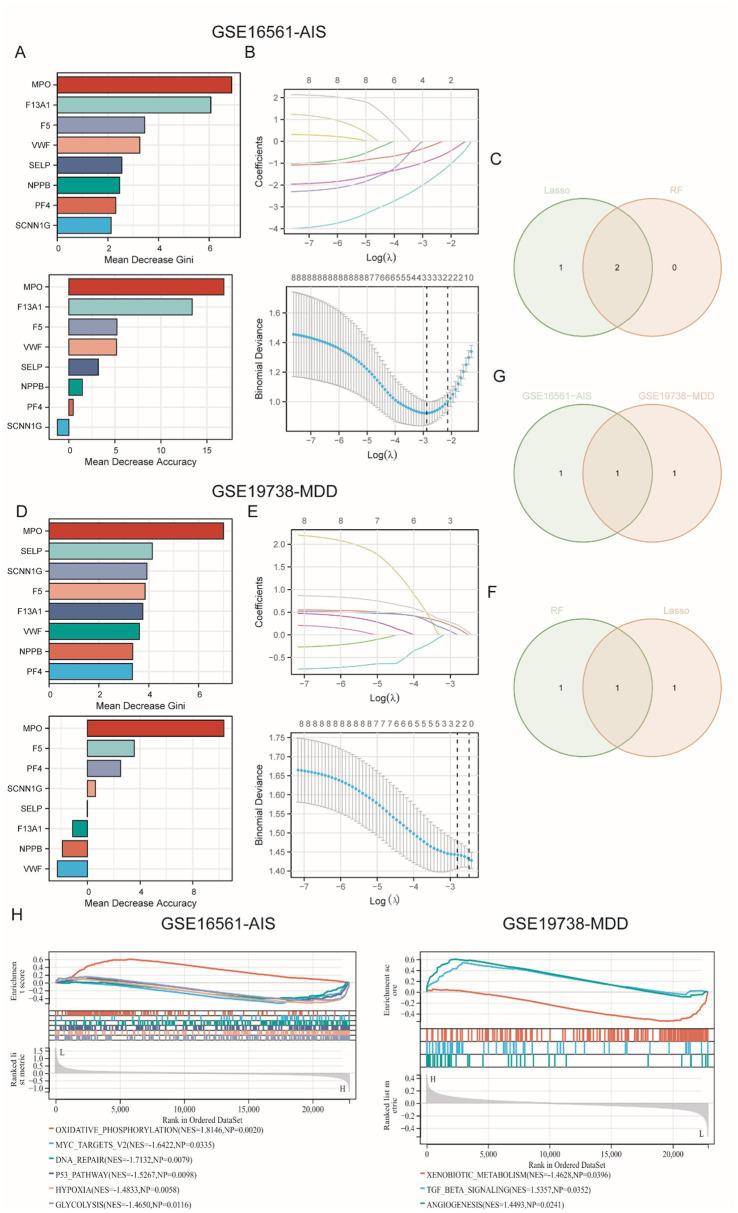
Identification of the hub gene MPO. **(A,B)** Variable importance plots from the RF and LASSO models in the AIS training set. **(C)** Identification of the TAM-related hub gene in the AIS training set. **(D,E)** Variable importance plots from the RF and LASSO models in the MDD training set. **(F)** Identification of the TAM-related hub gene in the MDD training set. **(G)** Identification of the shared TAM-related hub gene in the AIS and MDD training sets. **(H)** Single-gene GSEA of MPO in the AIS and MDD training sets.

A two-step machine learning pipeline (RF followed by LASSO regression) applied to the eight TAM-associated DEGs in both training sets consistently identified myeloperoxidase (MPO) as the top-ranking hub gene (Figures 3A–F). Single-gene GSEA revealed that high MPO expression was associated with oxidative stress and inflammation in both AIS and MDD contexts (Figure 3H).

### 3.4 Identification of the TNK target-associated diagnostic model for AIS and MDD patients

**Figure 4:**
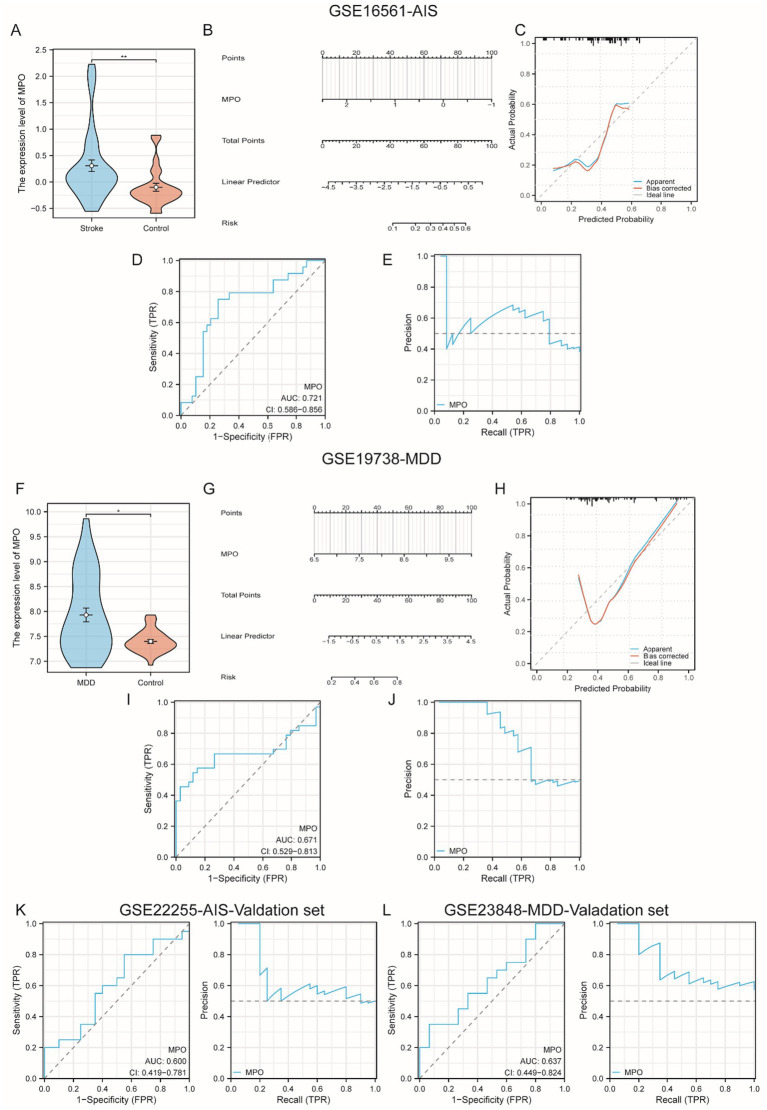
Diagnostic model construction and validation of MPO in AIS and MDD. **(A,F)** Violin plots of MPO expression comparing disease and control groups in the AIS and MDD training sets. **(B,C,G,H)** Nomograms and calibration analyses in the AIS and MDD training sets. **(D,E,I,J)** ROC and PR curve analyses of MPO in the AIS and MDD training sets. **(K,L)** ROC and PR curves of MPO in the AIS and MDD validation sets.

MPO expression was significantly upregulated in both AIS and MDD patients within the training sets (Figures 4A,F). It showed satisfactory diagnostic performance, with AUC values of 0.721 (AIS) and 0.671 (MDD) (Figures 4D,E,I,J). The nomograms and calibration curves demonstrated good predictive accuracy in both AIS and MDD cohorts (Figures 4B,C,G,H). These results were successfully validated in the independent cohorts GSE22255 (AIS, AUC = 0.60) and GSE23848 (MDD, AUC = 0.637) (Figures 4K,L).

### 3.5 Single-cell atlas and TNK target-associated hub gene distribution in AIS

**Figure 5:**
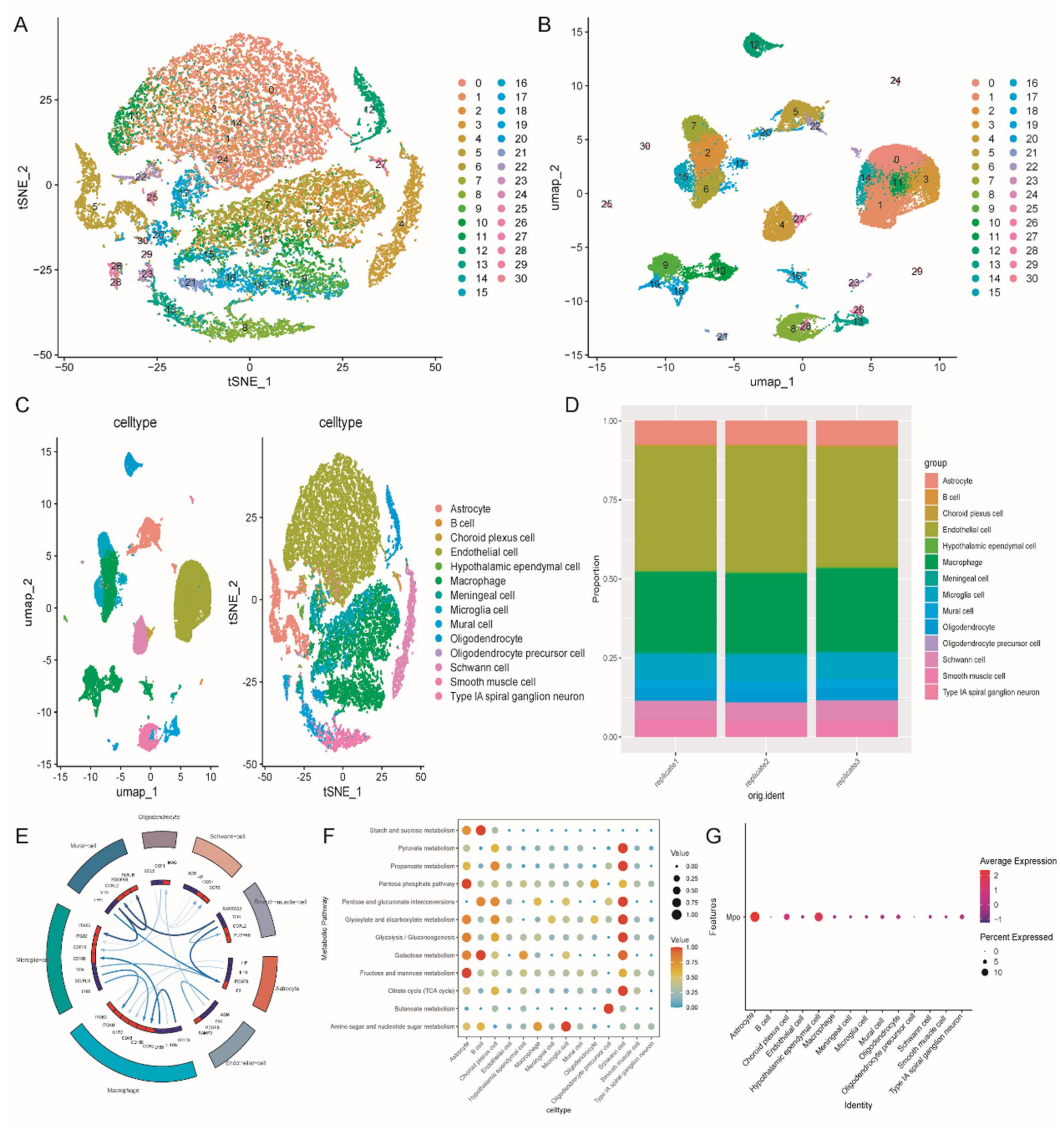
Single-cell landscape of the ischemic mouse brain. **(A)** t-SNE visualization of all cells colored by 30 annotated clusters. **(B)** UMAP visualization of all cells colored by 30 annotated clusters. **(C)** UMAP and t-SNE plots colored by major cell type annotations. **(D)** Stacked bar chart showing cell-type proportions across groups. **(E)** Circle plot of significant intercellular communication pathways. **(F)** Dot plot of metabolic pathway activity scores across cell types. **(G)** Dot plot showing Mpo expression across cell types.

Analysis of the AIS mouse brain scRNA-seq dataset identified 28,540 high-quality cells, which were clustered into 30 distinct cell populations (Supplementary Figure S1A–D and Figures 5A,B). Next, based on these clusters, we identified 14 cell types (Figures 5C,D). The CellChat analysis showed enhanced neuroinflammatory signaling of various cell types (Figure 5E). Metabolic profiling indicated disrupted metabolic pathways among these cell types (Figure 5F). Mpo expression was predominantly localized to the astrocyte cluster (Figure 5G).

### 3.6 Estimation of the TNK target-associated hub gene in MDD using AIS single-cell data

**Figure 6:**
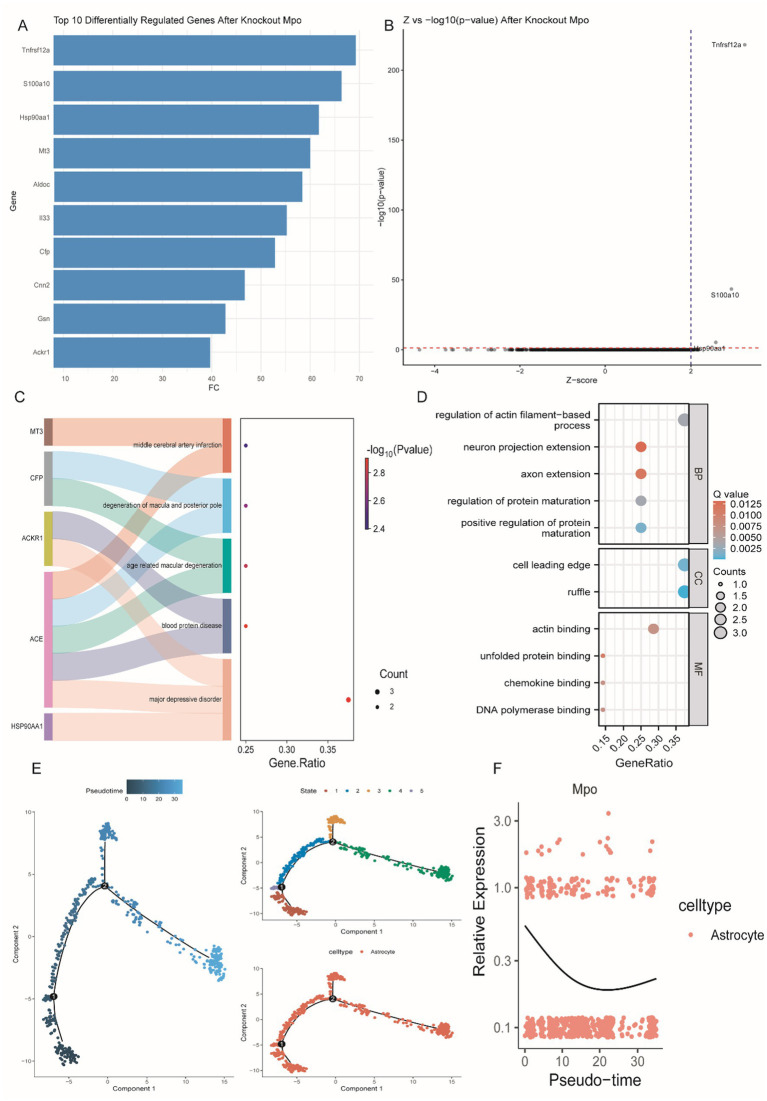
Functional implications of MPO in astrocytes. **(A,B)** Network graphs showing the top 10 genes perturbed by the virtual knockout of Mpo in astrocytes. **(C)** Bar plot of DO terms enriched for the perturbed genes. **(D)** Bubble plot of the top KEGG and GO pathways enriched for the perturbed genes. **(E,F)** Monocle 2 analysis.

The virtual knockout of Mpo specifically in astrocytes identified the top 10 significantly perturbed genes (Figures 6A,B). DO enrichment analysis of ACE and ACKR1 revealed an association with depressive disorder (Figure 6C). KEGG and GO analyses of the perturbed genes showed enrichment in functions related to neuroactive ligand–receptor interactions and the regulation of neurotransmitter levels(Figure 6D). Furthermore, the Monocle 2 analysis revealed five distinct differentiation patterns of astrocytes, indicating that Mpo expression decreases during astrocyte differentiation (Figures 6E,F).

### 3.7 Therapeutic screening for MDD in AIS patients

**Figure 7:**
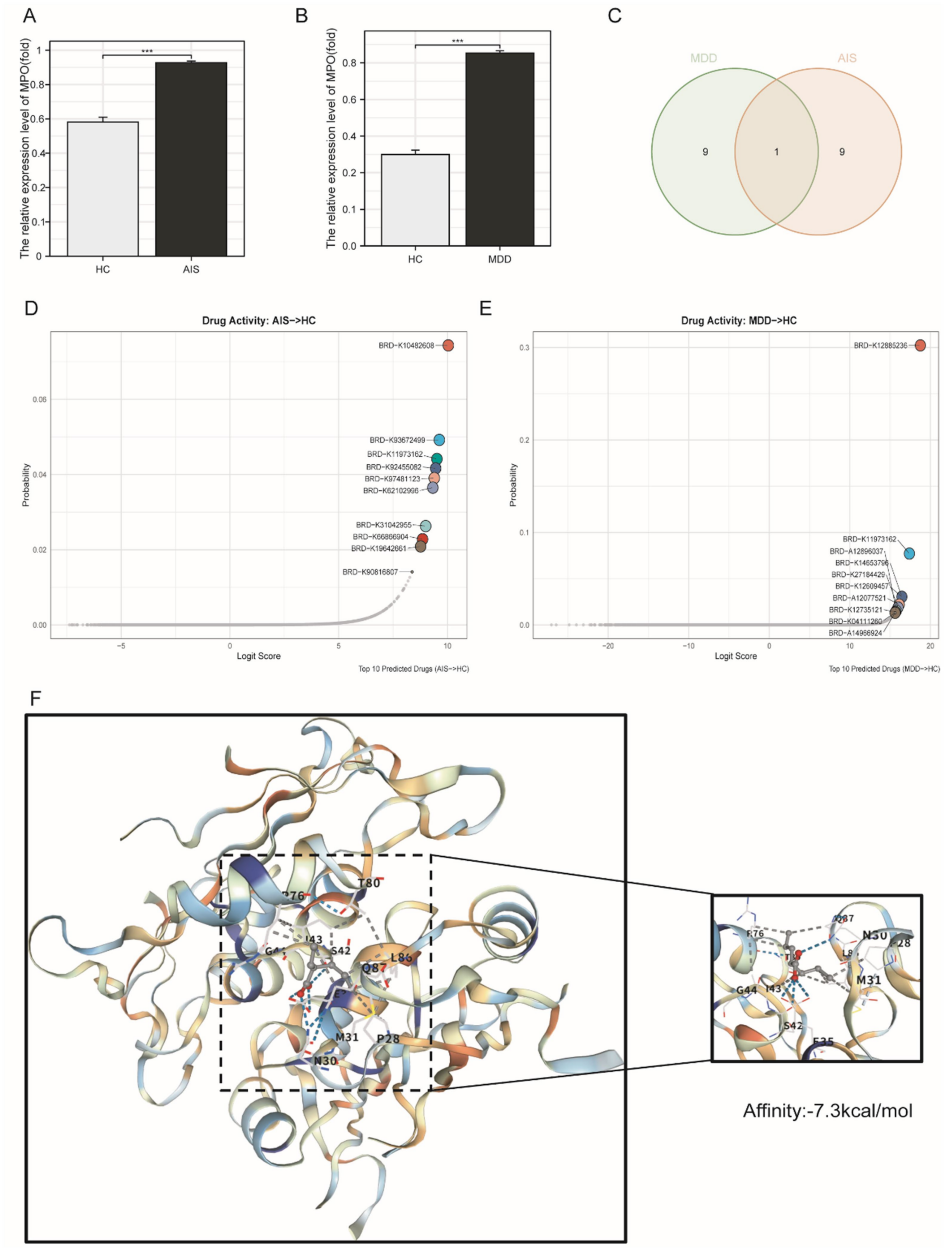
Therapeutic agent discovery and experimental validation. **(A,B)** qRT-PCR validation of MPO upregulation in the OGD/R and corticosterone models. **(C)** Venn diagram showing BRD-K11973162 as the intersection of the top drug candidate. **(D,E)** DrugReflector analysis in the AIS and MDD training cohorts. **(F)** Molecular docking estimation.

DrugReflector screening of the CMAP database based on the GSE16561 (AIS) and GSE19738 (MDD) signatures yielded separate candidate lists (Figures 7D,E). BRD-K11973162 was identified as the common top-ranking therapeutic candidate predicted to reverse both disease signatures (Figure 7C). Molecular docking predicted a stable binding conformation between BRD-K11973162 and the MPO protein, with a favorable binding affinity of −7.3 kcal/mol (Figure 7F). *In vitro*, MPO mRNA was significantly upregulated in HT22 neurons after OGD/R and corticosterone treatment (Figures 7A,B).

## 4 Discussion and conclusion

This study establishes a novel molecular framework that links TNK treatment to the risk of depression in AIS patients, with a focus on the hub gene MPO. Our multi-omics approach demonstrates that a TNK-target-associated gene signature stratifies patients into subgroups, identifies MPO as a potential cross-disease diagnostic biomarker, elucidates its astrocyte-specific role in promoting a depression-susceptible state, and nominates a promising therapeutic candidate for MDD pathogenesis in AIS patients following TNK treatment.

MPO is a heme-containing peroxidase abundantly expressed in neutrophils and is critical for generating microbicidal hypochlorous acid (Lin et al., 2024). In AIS, MPO released from infiltrating neutrophils exacerbates blood–brain barrier disruption, oxidative stress, and neuronal death (Fu et al., 2022; Wang et al., 2022). In addition to its classic role, a growing body of literature implicates MPO in neuropsychiatric disorders (Jannesar and Soraya, 2025). Elevated MPO levels and activity have been found in the serum and post-mortem brains of MDD patients, correlating with disease severity (Baranyi et al., 2022). MPO-generated oxidants can damage lipids and proteins, impair neurogenesis, and promote a pro-inflammatory milieu, all of which are implicated in depression pathophysiology (Li et al., 2026). In addition, an independent study illustrated that MPO levels are significantly decreased in patients following TNK treatment (Stewart et al., 2017). However, there are still gaps in understanding MPO patterns in the modulation of MDD pathogenesis in AIS patients after TNK treatment.

In conclusion, we have decoded an MPO-centric pathway that may explain the increased risk of depression following TNK therapy in AIS. This study identifies a tangible diagnostic biomarker (MPO) and a candidate therapeutic agent (BRD-K11973162), offering a novel mechanism-informed strategy to improve the long-term neurological and psychiatric outcomes of stroke survivors. However, this study has inherent limitations that define clear directions for future research. First, the diagnostic and therapeutic potential of MPO, derived from *in silico* analyses, must be prospectively validated in preclinical and clinical studies involving TNK-treated patients or experimental models of AIS with MDD pathogenesis. Second, the TNK-target-associated mechanistic role of MPO in astrocytes contributing to MDD pathogenesis in AIS patients, inferred from single-cell analysis and virtual knockout, requires direct experimental validation in models of AIS with MDD pathogenesis. In addition, studies employing astrocyte-specific Mpo knockout or inhibition in AIS mouse models with MDD after TNK treatment, combined with behavioral assessments, are essential to establish causality and elucidate downstream molecular pathways. Furthermore, for the TNK-target-associated therapeutic candidate, preclinical and clinical studies should be conducted to evaluate its molecular mechanisms in the pathogenesis of MDD in AIS patients. Furthermore, the therapeutic effects of BRD-K11973162 on the inhibition of MDD in AIS patients, and whether MPO is the target of BRD-K11973162, should be evaluated in preclinical and clinical studies to assess therapeutic efficacy and safety. Finally, the clinical relevance of the identified TAM signature and MPO should be examined in human post-mortem brain tissue from AIS patients with and without comorbid depression.

## Data Availability

Publicly available datasets were analyzed in this study. These data are available in the Gene Expression Omnibus under accession numbers GSE58294, GSE32280, GSE16561, GSE19738, GSE22255, GSE23848, and GSE174574. The TNK target gene list is provided in Supplementary Table S1.
