# Supplementary figures and images for: Assessment of tenecteplase target-associated pathogenic mechanisms underlying depression in acute ischemic stroke patients: insights from artificial intelligence-driven multi-omics analysis and in vitro validation
Source: Front Neurosci. 2026 Jun 16;20:1848128. doi: 10.3389/fnins.2026.1848128 (PMC13315191; doi:10.3389/fnins.2026.1848128)

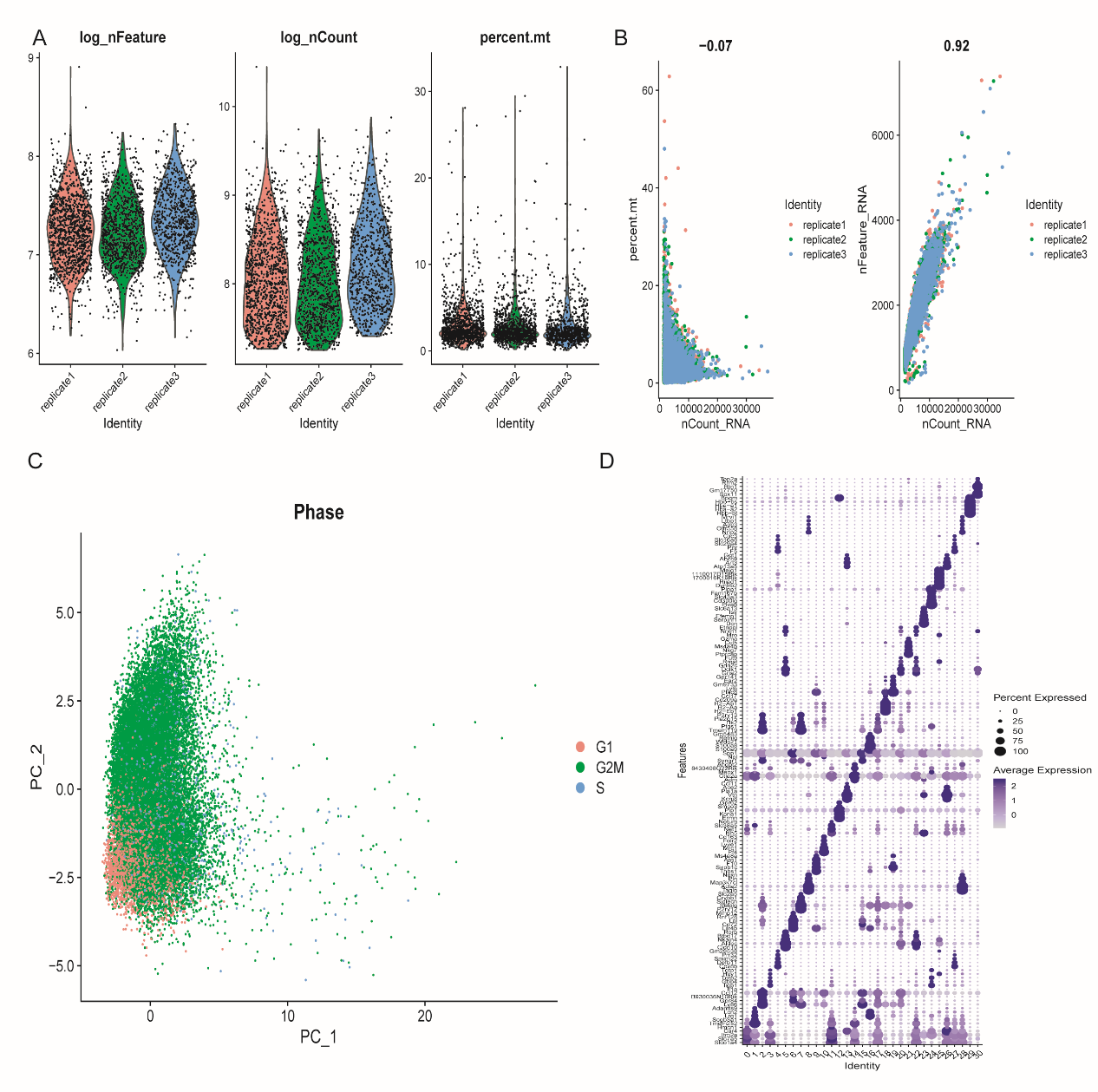


**Figure S1: Pre-processing of single-cell data.** (A-C) QC metrics. (D) Markers of clusters.

Supplement: Supplementary file 1 [file Data_sheet_1.docx]
